# Effects of self-reported sensitivity and road-traffic noise levels on the immune system

**DOI:** 10.1371/journal.pone.0187084

**Published:** 2017-10-30

**Authors:** Ahra Kim, Joo Hyun Sung, Jin-Hee Bang, Seung Woo Cho, Jiho Lee, Chang Sun Sim

**Affiliations:** 1 Environmental Health Center, University of Ulsan College of Medicine, Ulsan, Republic of Korea; 2 Department of Occupational and Environmental Medicine, Gyeongsang National University Changwon Hospital, Gyeongsang National University School of Medicine, Changwon, Republic of Korea; 3 Department of Occupational and Environmental Medicine, Ulsan University Hospital, University of Ulsan College of Medicine, Ulsan, Republic of Korea; Boston University, UNITED STATES

## Abstract

Sensitivity to noise, particularly road traffic noise, can increase cortisol levels and result in changes in immune system biomarkers. Therefore, continuous exposure to noise can have an effect on immune function, hormonal levels, and cardiovascular function, leading to hypertension and stress. The purpose of this study was to investigate the changes in stress-and immune system-related biomarkers according to the self-reported sensitivity to noise and exposure to road traffic noise, to ultimately determine the potential effects of noise on health. A survey was conducted through questionnaire (ISO/TS 15666) sent to 172 female subjects in Korea, including 128 from Ulsan and 44 from Seoul. The average noise level was calculated, and blood samples were collected for measurements of cortisol levels, Natural killer (NK) / Natural killer T (NKT) cell populations, and NK cell activity (through measurements of interleukin-12 (IL-12) and interferon-gamma (INF-γ) concentrations). Multivariate linear regression analysis of the measured biomarkers according to the road traffic noise level and self-reported noise sensitivity was conducted adjusting for the effects of age, alcohol status, smoking status, regular exercise, and residence period. IL-12 levels increased, whereas the NKT cell population decreased with increasing noise levels. The results further suggested that cortisol levels are more influenced by the subject’s sensitivity to noise than to the level of chronic road traffic noise. Therefore, noise appears to have the largest effect on IL-12 levels as well as the population and activity of NKT cells. In conclusion, our results suggest that low-level road traffic noise and sensitivity to noise can affect health by causing changes in the immune response through mechanisms other than increased cortisol.

## Introduction

Environmental noise can be defined as the noise emitted from all sources, other than noise at an industrial workplace [1]. Several studies have shown that environmental noise such as that due to traffic, aircrafts, and construction can have physiological and psychological consequences [2, 3] such annoyance, sleep disturbance [4], cardiovascular disease [5], hypertension [6] and stress [7, 8]. Therefore, there has been much research interest on the potential health effects of exposure to environmental noise.

Through meta- analysis, a small number of studies have shown that noise-induced cardiovascular disease is associated with a higher risk for men than for women and a higher risk for people aged older than 65 years [9].

Recent studies have also reported that sleep quality and noise sensitivity are not related to vascular function or noise sensitivity, but rather that night noise increases the risk of cardiovascular disease due to increased blood pressure in patients and controls [10]. Road traffic noise is a particular noise source that affects a large portion of urban populations. Indeed, more than 40% of the populations in European Union countries are exposed to noise of 55 dB or above; 20% are exposed to daytime noise of 65 dB and above, and more than 30% are exposed to nighttime noise of 55 dB or above [1]. The World Health Organization stated that noise has negative effects on health by triggering physiological changes such as impairment of hearing function and increases in stress hormones and sensitivity [11, 12].

The most well-known mechanism mediating the response to such stress is the hypothalamic-pituitary- adrenal axis (HPA axis). When the HPA axis receives a signal of a stress response, corticotropin releasing factor is secreted from the hypothalamus, releasing adrenocorticotropic hormone from the pituitary gland. Adrenocorticotropic hormone then promotes the secretion of cortisol from the adrenal cortex through the blood, which triggers responses to various kinds of stress. The secretion of cortisol in response to stress inhibits the function of the HPA axis to disrupt the secretion of neurohormones and neurotransmitters as well as influencing the endocrine system, thereby disturbing homeostasis of the body, which can induce the development of various stress-related diseases[13]. Recently Meyer et al. [14] reported that mental stress and noise exposure could activate inflammatory cytokines such as interleukin (IL)-6 and IL-1β. These cytokines also interact with each other. Extracllular IL-6 induces cortisol from the zona fasciculate of the adrenal cortex, and has been reported to affect the synthesis of cortisol even when the HPA axis is inhibited. Thereby, the immune, endocrine and nervous systems are related to each other.

Thus, environmental noise has direct effects on health as well as indirect effects through the release of stress hormones such as cortisol [15, 16], dopamine [17, 18] and changes of alpha-amylase levels [19]. Increased cortisol can further cause changes in the immune system such as cellular proliferation, cytokine secretion, antibody production and cytotoxicity [20, 21]. Indeed, one study showed that the activity of natural killer (NK) cells was decreased by increased cortisol [22]. NK cells are a leukocyte subset and important components of innate immunity. The innate immune system provides a rapid, non-specific host response against foreign agents such as bacteria, viruses, or tumors before triggering the adoptive immune system [23–25]. Innate immunity includes the antigen-presenting cells, monocytes/macrophages, dendritic cells (DCs), NK cells, and NKT cells.

Moreover, cytokine signaling is essential for intercellular communication in the immune system to mediate and control immune functions. IL-12 is an important cytokine mediating immune responses, which is mainly produced by monocytes, macrophages, and DCs in response to bacterial products, intracellular pathogens, or upon interaction with activated T cells. IL-12 has been shown to play a critical role in the pathogenesis of a variety of immune-related diseases. This cytokine can induce interferon-gamma (IFN-γ) production [26], cell proliferation, and cytotoxicity mediated by NK cells and T cells.

Hence, exposure to environmental noise affects immunity by inducing stress and increasing the secretion of stress hormones. As sensitivity to noise itself will affect the stress response and such sensitivity varies among individuals, the effects and response of exposure to identical environmental noise can show high individual variation.

However, studies on the negative effects of environmental noise, including road traffic noise, on health are still lacking in Korea. Therefore, the aim of our study was to analyze the immune response against noise generated from road traffic as well as the self-reported sensitivity to noise in two metropolitan cities in Korea. Specifically, the level of exposure to road traffic noise was estimated using the noise map generated for Ulsan Nam-gu and Seoul Yangchun-gu, and the changes of immune response parameters according to road traffic noise and sensitivity were statistically analyzed.

## Material and methods

### Participants and noise measurement

All subjects participated voluntarily and provided written informed consent.

We recruited 1,000 people in Yangcheon-gu (Seoul) and Nam-gu (Ulsan), respectively from July 2015 to December 2015. Participants were stratified according to noise exposure level based on noise map data. Although the residents of Yangcheon-gu are exposed to aircraft and road traffic noise, those of Nam-gu are exposed only to road traffic noise.

**Fig 1:**
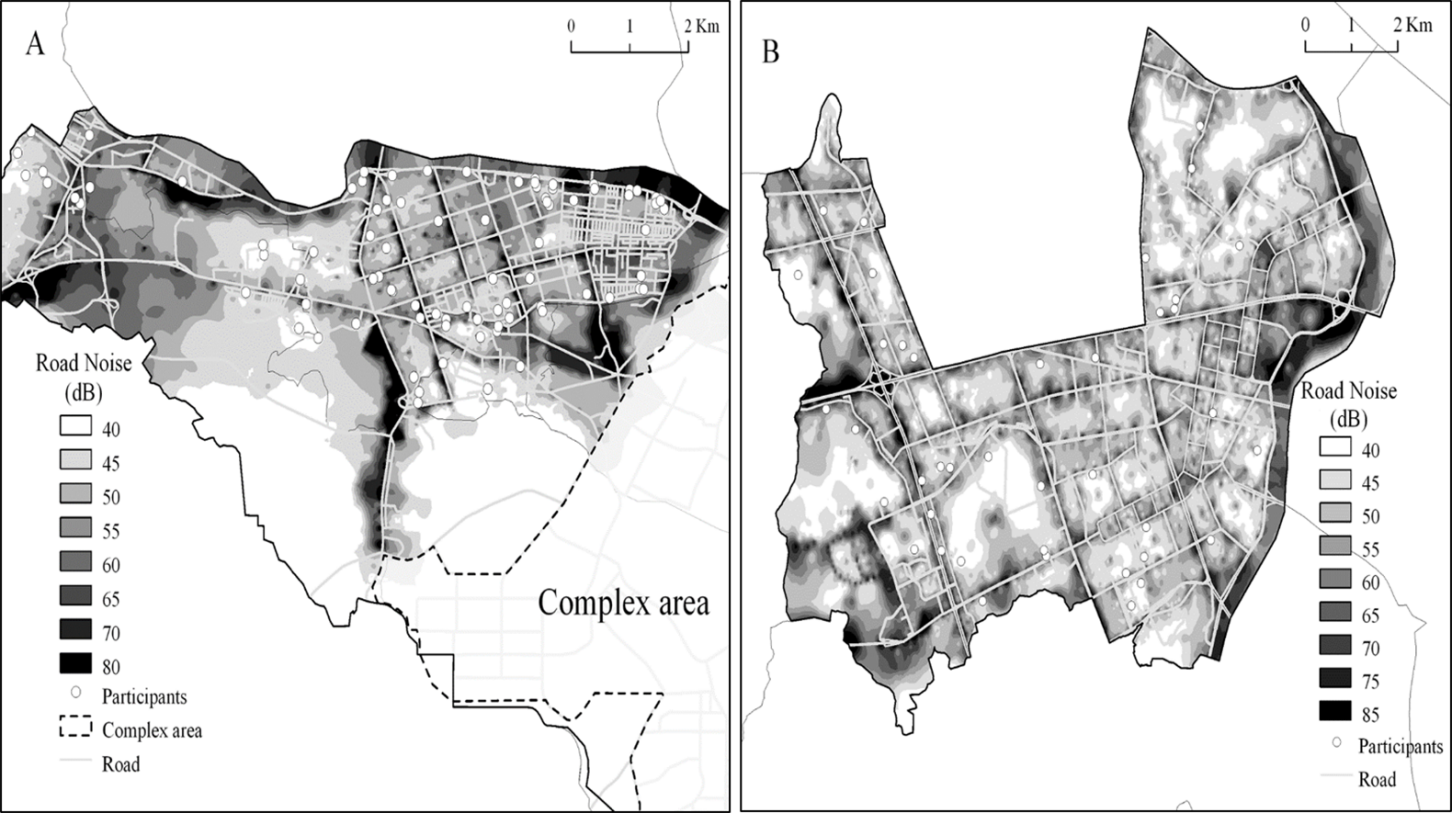
**Location of residents in Ulsan (A, Nam-gu), Seoul (B, Yangcheon-gu) in noise map.** It shows the noise level of each area and the location of participants in Nam-gu of Ulsan (A) and Yancheon-gu of Seoul (B).

A total of 336 participants each in Nam-gu, Ulsan and Yangcheon-gu, Seoul finally agreed to the blood test and included in the study for analysis (Fig 1).

The questionnaires and blood test results of 336 participants were reviewed. Questionnaires were missing for four subjects, 93 subjects were excluded because of underlying chronic diseases, and 67 subjects were men; thus, 172 female subjects were finally selected.

The male participants were excluded because they tend to have more activity in environments outside of the residence target area, which could influence the sensitivity to the environmental noise around the residence. Subjects with any chronic illness (hypertension, hyperlipidemia, stroke, myocardial infarction, angina, arrhythmia, diabetes, and other diseases) were also excluded, because of a potential interference on the influence of noise on the immune response.

To examine the level of environmental noise that the participants were exposed to, a three-dimensional noise map generated in 2014 based on the participants’ addresses confirmed from the survey was used to calculate the mean noise levels of the buildings the participants reside in as a road traffic noise index.

The noise index used in this study was the day-night average sound level (Ldn), which is a qualitative index of the weighted equivalent day/night noise level that divides a 24h period into 6 am–10 pm and nighttime.

Actual noise exposure levels were assessed using a sound level meter (Ulsan: NA-28, Rion, Japan; Seoul: B&K 2250, Brüel & Kjær, Denmark).

### Questionnaires

The questionnaires included age, residence period, education level, monthly income, alcohol status, smoking status, and exercise status. Education level was divided into a high school degree or below and a community college degree or above. Monthly income was divided into less than 3 million Won and above. Smoking status was divided into current smokers and current non-smokers (past smokers and non-smokers). Alcohol status was divided into current drinker and current non-drinker (past drinker and non-drinker). Exercise status was divided into participants currently exercising regularly and those who were not. To assess the sensitivity to noise, an 11-point visual analogue scale was generated and used in this study based on ISO/TS 15666 [27].

All research procedures were approved by the Ulsan University Hospital Institutional Review Board (UUh 2014-08-008-012).

### Blood sampling and NK cell preparation and flow cytometry

After the subjects were selected, 7 mL of venous whole blood was taken from the respondents between 9.00 am to 12.00 pm.

Peripheral blood mononuclear cells (PBMCs) were isolated from all 172 subjects. The NK cell population was analyzed by fluorescence-activated cell sorting (FACs) at Green Cross LabCells Corporation (Seoul, Korea) to identify the proportion of CD45^+^CD16^+^CD56^+^ cells. Cortisol levels were measured by Quantikine enzyme-linked immunosorbent (ELISA) analysis using serum from Green Cross LabCells Corporation. The concentrations of IL-12 and INF-γ in the sera were measured using the Quantikine enzyme-linked immunosorbent (ELISA) kit, according to the manufacturer's instructions (Bio Legend San Diego, CA, USA).

### Statistical analysis

The statistical analysis was performed with SPSS v.21 for Window (IBM SPSS Inc., Chicago, IL, USA). The data regarding immune response parameters such as cortisol, NK cell population, and cytokines showed positively skewed distributions; thus, logarithmic transformations were performed for these variables to facilitate further statistical analyses under normal data assumptions.

Statistical analyses were performed using multiple linear regression and Pearson’s correlation analysis. The results were considered statistically significant when P < 0.05.

## Results

The sociodemographic variables and sensitivity to noise for 128 participants in Ulsan Nam-gu and the 44 participants in Seoul Yangchun-gu are summarized in Table 1.

**Table 1:** General characteristics of the subjects.

			Ulsan			Seoul			Total		
			N	Mean	95% CI	N	Mean	95% CI	N	Mean	95% CI
Age (years)			128	44.9	42.9–46.8	44	45.6	41.8–49.5	172	45.1	43.3–46.8
		20–29 years	11 (8.6)			5 (11.4)			16 (9.3)		
		30–39 years	29 (22.7)			7 (15.9)			36 (20.9)		
		40–49 years	40 (31.3)			14 (31.8)			54 (31.4)		
		50–59 years	39 (30.5)			13 (29.5)			52 (30.2)		
		Over 60 years	9 (7.0)			5 (11.4)			14 (8.1)		
Residence period (years)			128	9.6	8.3–10.9	42	7.9	5.5–10.3	170	9.2	8.0–10.3
Noise sensitivity			128	5.4	5.1–5.8	44	6.5	5.9–7.2	172	5.72	5.38–6.05
Education level		High school and less	62			23			85 (49.4)		
		College and more	66			21			87 (50.6)		
Income		Under 3,000,000 (KWR)	16			19			35 (20.3)		
		Over 3,000,000 (KWR)	112			25			137 (79.7)		
Alcohol status		No	48			28			76 (44.2)		
		Yes	80			16			96 (55.8)		
Smoking status		No	125			44			169 (98.3)		
		Yes	3			0			3 (1.7)		
Regular exercise		No	70			12			82 (47.7)		
		Yes	58			32			90 (52.3)		

Unit: Number (percentage)

In both Ulsan and Seoul, the distribution rate of participants was highest at 31.3% and 31.8%, respectively. The average age of all participants in Ulsan was 42.9 years and the average age of all participants in Seoul was 45.6 years. The age group with the lowest participation rate was more than 60 years in Ulsan and Seoul. In the case of Seoul, the subjects in their 20s also showed the lowest participation rate.

The mean age of the participants was 45.1 years and the mean residence period at the current residence was 9.2 years. The mean sensitivity to noise was 5.72. With respect to education level, 49.4% of the participants had a community college degree or below and 50.6% had a community college degree or above. For monthly income, 79.7% of the participants reported earning 3,000,000 Won or more and 20.3% reported earning less than 3,000,000 Won. The frequency of current and non-drinkers was 55.8% and 44.2% respectively, and the frequency of current and non-smokers was 1.7% and 98.3%, respectively. In addition, 52.3% of the participants reported exercising regularly and 47.7% reported that they did not exercise regularly.

**Fig 2:**
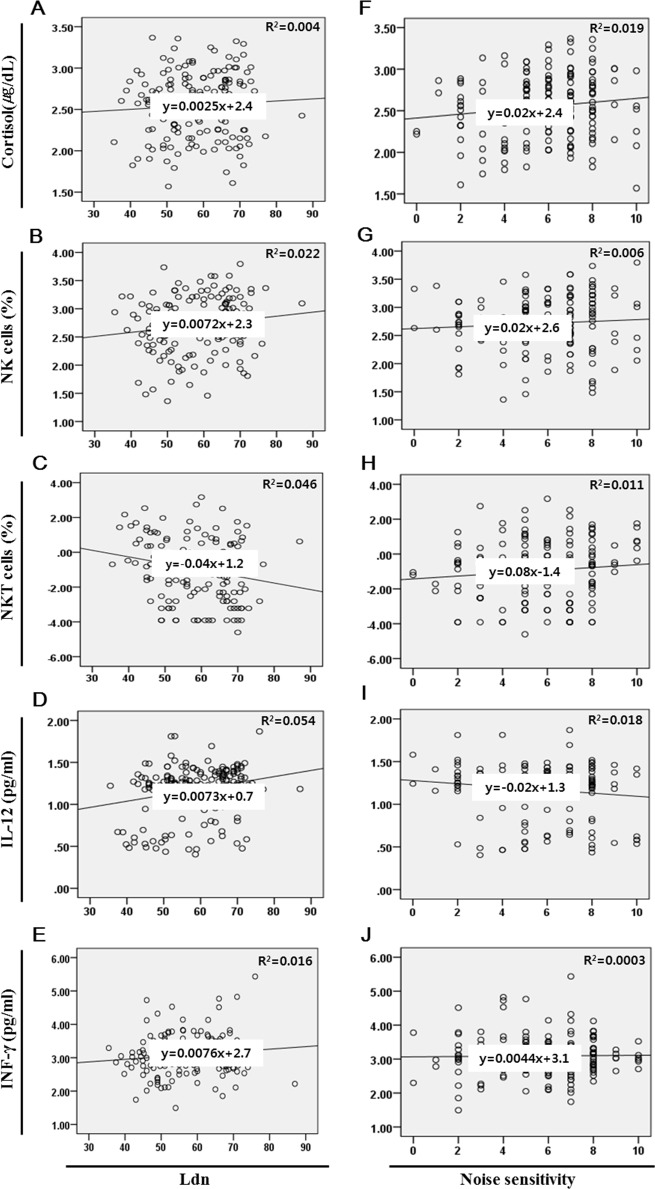
Each real correlation curve for biomarker, Ldn and noise sensitivity. (A-E) It shows each the real correlation curve for Ldn and biomarker such as cortisol (A), NK(B) and NKT cells (C), cytokines (D and E)) and it also shows each the real correlation curve for noise sensitivity and biomarker(F-J).

As shown in Table 2, road traffic noise and IL-12 were significantly positively correlated (r = 0.2333). Road traffic noise and the NKT cell distribution rate showed a significant negative correlation (r = 0.214; P < 0.01). IL-12 and the NKT distribution were also negatively correlated (r = 0.622), whereas IL-12 and INF-γ showed a significant positive correlation (P < 0.01). The change in biomarker for Ldn and noise sensitivity are shown using correlation curves in Fig 2.

**Table 2:** Correlation among noise levels, noise sensitivity and immune response.

Variables	Ldn	Noise sensitivity	Cortisol	NK cells	NKT cells	IL-12
**Noise** **sensitivity**	-0.103					
**Cortisol**	0.065	0.138				
**NK cells**	0.147	0.074	0.057			
**NKT cells**	-0.214**	0.106	-0.138	-0.120		
**IL-12**	0.233**	-0.134	0.149	0.007	-0.620**	
**INF-γ**	0.127	0.017	0.041	-0.167	-0.148	0.251**

**. Correlation is significant at the 0.01 level (2-tailed), Immunologic profiles were transformed by natural log.

**Fig 3:**
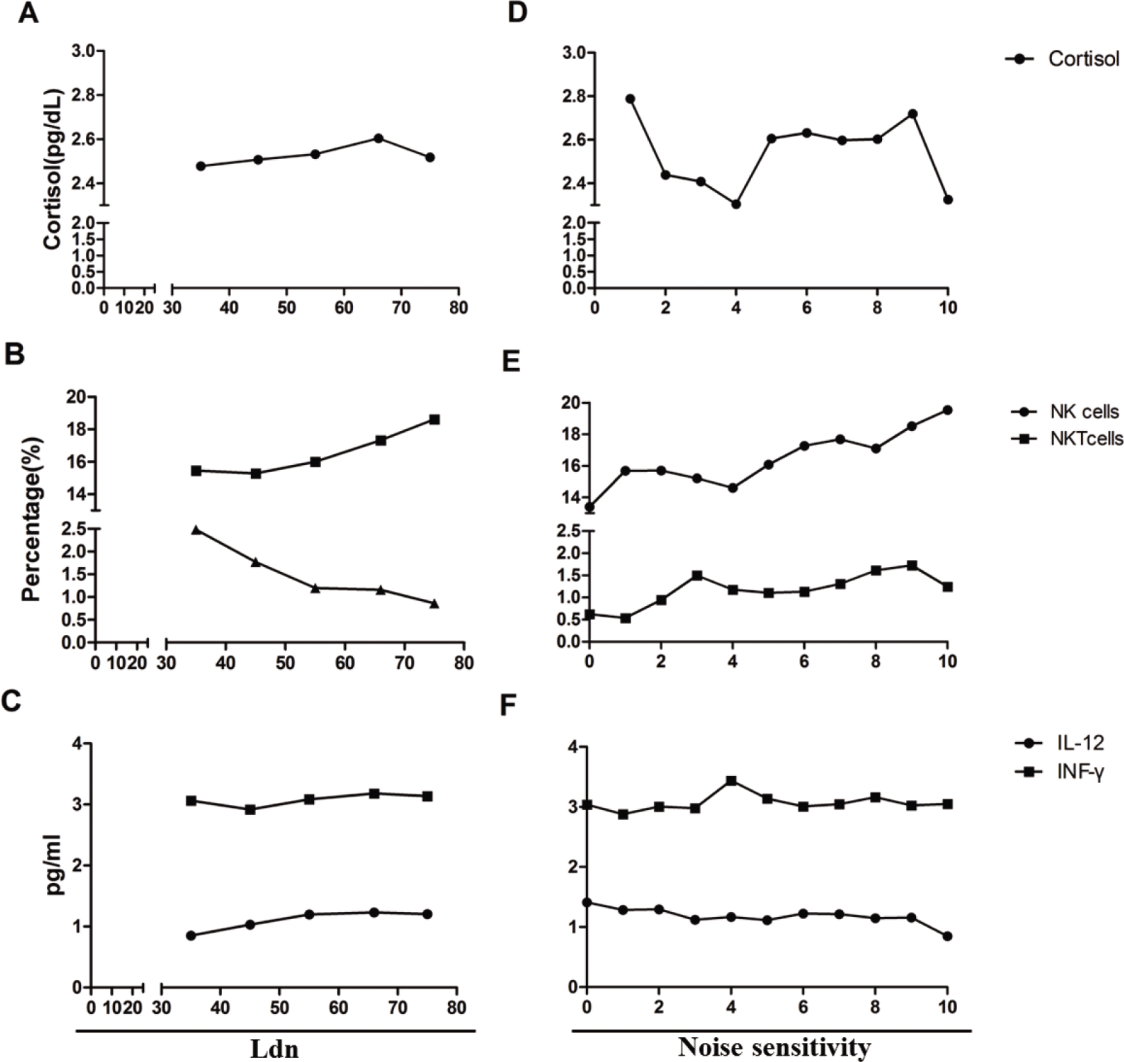
Adjusted predicted value change of biomarker for Ldn and noise sensitivity interval. The values of cortisol (pg/dL), NK and NKT cells (%), cytokines (pg/ml) were adjusted for age, alcohol consumption, smoking status, regular exercise, residence period. The mean values of cortisol (A), NK/NKT cells (B), cytokines (C) for each interval for Ldn are shown, and D-F shows the mean value of each biomarker for the noise sensitivity interval.

Multivariate linear regression analysis was performed to analyze the relationships among road traffic noise, individual sensitivity, and age-dependent biomarkers, adjusting for the effects of alcohol status, smoking status, regular exercise, and residence period. As shown in Table 3, as the sensitivity level increased by 1 step, the cortisol level increased by 0.032 μg/dL. Moreover, as the road traffic noise increased by 1 dB, the percentage of NKT cells decreased by—0.038%, whereas the IL-12 level increased by 0.006 pg/mL. The percentage of NK cells and INF-γ levels were not significantly associated with road traffic noise or sensitivity (P > 0.05), and therefore were meaningless as regression coefficients. As shown in Fig 3, the mean value of Ldn and the noise sensitivity interval are shown as correlation curves through the adjusted predicted value of each biomarker.

**Table 3:** Immune responses to road traffic noise, noise sensitivity, and stress response.

Variables		Multivariate analysis		
Dependent	Independent	β±SE	p-value	R^2^
ln_Cortisol (㎍/dL)	Ldn	0.003±0.003	0.319	0.057
	Sensitivity	0.032±0.014	0.020	
ln_NK cells (%)	Ldn	0.006±0.004	0.111	0.193
	Sensitivity	0.018±0.016	0.263	
ln_NKT cells (%)	Ldn	-0.033±0.014	0.021	0.079
	Sensitivity	0.046±0.063	0.464	
ln_IL-12 (pg/mL)	Ldn	0.006±0.002	0.010	0.097
	Sensitivity	-0.010±0.011	0.365	
ln_INF-γ(pg/mL)	Ldn	0.007±0.005	0.118	0.105
	Sensitivity	0.007±0.020	0.750	

SE: standard error, Immunologic profiles were transformed by natural log. Linear regression models adjusted for age, alcohol consumption, smoking status, regular exercise, residence period.

## Discussion

In this study, the correlations among road traffic noise, sensitivity to noise, stress hormones, and immunity-associated factors were investigated.

Previous studies have shown that a high level of noise or sensitivity to noise induces sleep disorders [28, 29], hypertension [30–32], and cardiovascular disease [33, 34].

The result of traffic noise, depression, and anxiety are somewhat limited and somewhat controversial; however, Beutel et al. [35] found that strong noise discomfort was related to higher depression and anxiety in the general population. Participants with a mental health problem may also have higher noise sensitivity and report higher discomfort levels [36].

When exposed to heavy nighttime aircraft, patients with coronary heart disease reported chest pain caused by a typical heart disease [37].

Moreover, these consequent increases in the levels of the stress hormone cortisol from noise or sensitivity can have negative effects on health by reducing the activity of NK cells [38, 39].

Duggal et al. [40] suggested that the observed association between NK cell immunesenescence and cortisol was more strongly affected by mental stress rather than by physical stress.

Munzel et al. [41] suggested a mechanism of the noise-triggered activation of the immune system using a mouse model, indicating that noise exposure not only increased levels of noradrenalin (NA), adrenalin (A) and angiotensin II (Ang II), but also increased cortisol thereafter. Increased Ang II has been reported to activate endothelial NADPH oxidase, which causes oxidative stress that can lead to direct scavenging of nitric oxide and enhanced nitric oxide synthatse uncoupling. Since then, the increase in the NADPH oxidase subunit NOX-2 has been reported to increase immune cell activation and infiltration, such as NK cells, myelomonocytic cells, leukocytes, and macrophages/monocytes.

Therefore, noise stress causes blood pressure and vascular dysfunction associated with oxidative stress.

In the present study, the IL-12 and INF-γ levels were positively correlated, suggesting that an increase of cortisol decreases the IL-12 level with a consequent decrease of NK cells and INF-γ, a marker of NKT cell activation.

Hence, it is predicted that an increase in cortisol levels would reduce immune function by decreasing the activity of NK cells and NKT cells.

Although there are few studies on the relationship between NKT cells and noise, these cells can be activated in both antigen-dependent and independent manners. Furthermore, because they have pro-inflammatory and immunoregulatory characteristics, they are known to play an important role in autoimmune diseases [42, 43], viral infections [44, 45] and cancer [46].

In this study, the level of road traffic noise showed a positive correlation with IL-12 levels but did not affect the NK cell population. However, the proportion of NKT cells was negatively correlated with road traffic noise.

In addition, multivariate linear regression analysis on the relationships between road traffic noise level or noise sensitivity to immune and stress parameters, controlling for the potentially confounding variables of age [47–49], alcohol status, smoking status, regular exercise, residence period, showed that the increase of noise level increased the IL-12 level but decreased the NKT cell distribution rate, similar to results of the correlation analysis. Because cortisol is considered to be more strongly affected by sensitivity than chronic road traffic noise and road traffic noise does not largely affect cortisol unlike the biological response to > 80 dB noise exposure [15], it appears that the IL-12 level and NKT cell frequency and activity are likely regulated by different mechanisms [15]. That is, cortisol increases through the HPA-axis, which is stimulated by a sensitivity response to activate DCs and macrophages, and the chronic noise itself can increase the levels of IL-12, a pro-inflammatory cytokine, as a synergistic effect. However, noise stress will decrease the NKT cell population to induce a pro-inflammatory response, with negative consequences for health [18].

The limitations of this study are as follows. First, as a cross-sectional study, although correlations could be determined, the cause-and-effect relationship cannot be established from these data. Second, the effects of extreme noise levels such as occupational noise were not considered. Finally, not all potentially confounding variables were controlled in the analysis.

Nevertheless, the clear associations detected in the present study and previous work suggest the importance of carrying out longitudinal studies on the effects of chronic noise on health and to determine the mechanism underlying the immune response to chronic loud noise. Although previous health effect evaluations associated with noise were mostly based on survey results, heart rate variability, stress hormones (e.g., cortisol, norepinephrine, epinephrine), or studies of the activation of NK cells in response to loud noise and noise sensitivity through *in vitro* experiments, clinical studies on a large number of subjects of similar ages should be performed to more objectively analyze the *in vivo* effects of exposure to a low level of environmental noise with respect to the involvement of NK cells, and NKT cells population and activation. Such investigations are expected to provide better indices for future health effect evaluations on the immune response against noise and sensitivity.

## Conclusions

**Fig 4:**
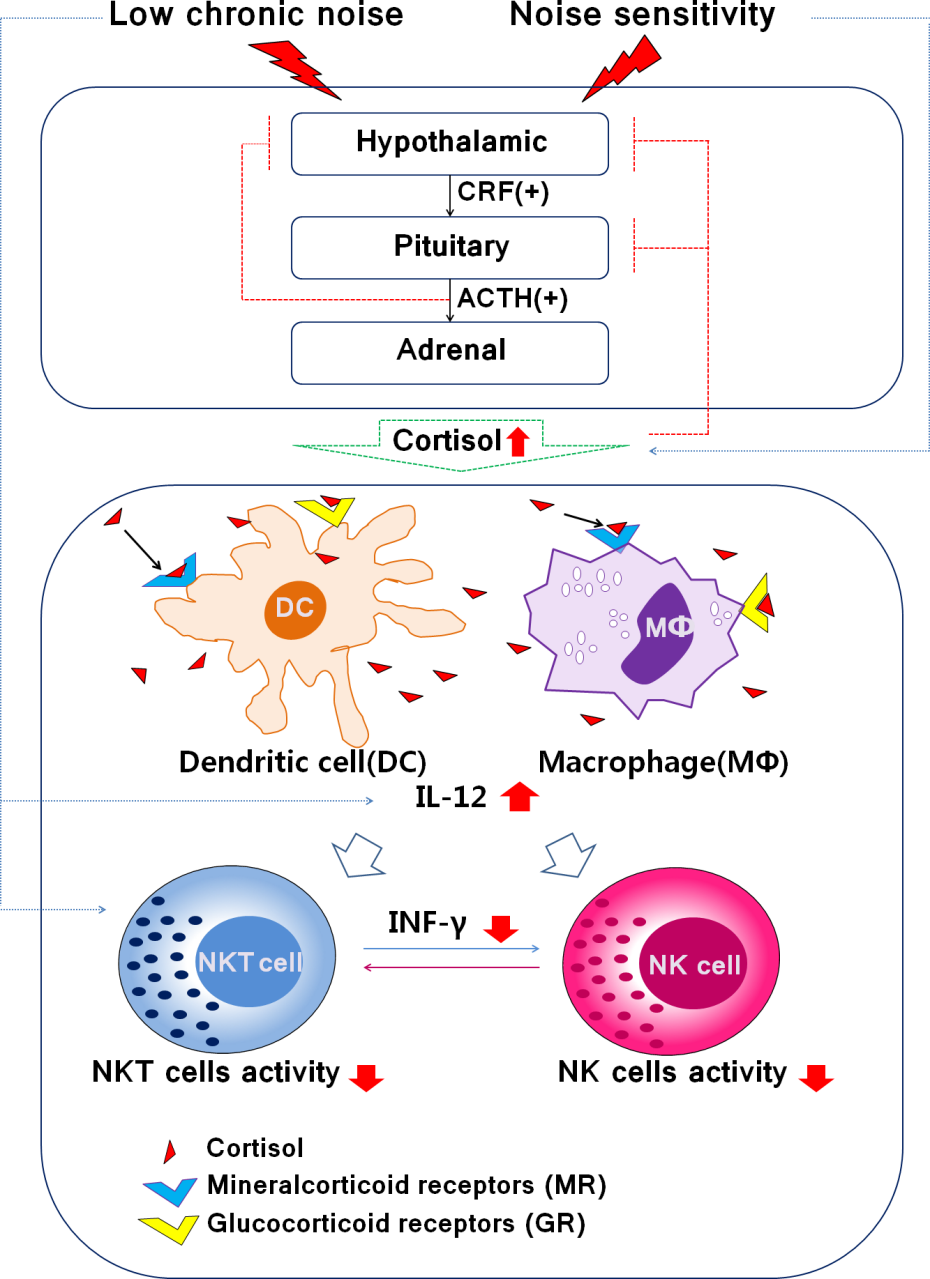
Schematic representation of the potential immune response by exposure noise levels or noise sensitivity. Low chronic noise affects DCs and Macrophages and increases IL-12 concentration, but noise sensitivity increases the concentration of cortisol in the body. Therefore, we expect to decrease INF-γ activity, NKT and NK cells population by Ldn and noise sensitivity, which are two influencing factors.

Low-level environmental noise and sensitivity to noise are likely to have negative effects on health by triggering changes in the immune response through a mechanism distinct from an increase in cortisol (Fig 4).

## Supporting information

S1 Dataset Full dataset of tables. (PDF)
