# Supplementary material for: Effects of self-reported sensitivity and road-traffic noise levels on the immune system
Source: PLoS One. 2017 Oct 30;12(10):e0187084. doi: 10.1371/journal.pone.0187084 (PMC5662213; doi:10.1371/journal.pone.0187084)
Supplement: S1 Dataset — (PDF) [file pone.0187084.s001.pdf]

Using sample	Blood test	ID	Region	Noise_Ldn	Age	Sex	Noise_sensitivity	Education	Income	Alcohol	Smoking	regular exercise	Residence period(year)	SRI_MF	Cortisol	NK cells	NKT cells	IL12	INFgamma
Y	Y	u-1	ulsan	65.00	36	male	1	high school and less	under 3000 KRW	1.00	1.00	2.00	8.00	0.00	10.70	26.30	0.1	3.566108957	26.63265306
Y	Y	u-2	ulsan	58.00	42	female	6	999	3000 KRW and more	2.00	1.00	2.00	9.00	0.00	11.60	13.40	0.1	4.183431953	27.00510204
Y	Y	u-3	ulsan	47.00	51	female	3	high school and less	3000 KRW and more	1.00	1.00	2.00	16.00	0.00	11.80	15.00	0.77	3.230769231	9.234693878
Y	Y	u-4	ulsan	51.00	24	female	5	college and more	under 3000 KRW	2.00	2.00	1.00	6.00	7.00	22.00	5.50	0.06	3.615792695	20.54591837
Y	Y	u-5	ulsan	67.00	57	female	7	high school and less	3000 KRW and more	1.00	1.00	1.00	19.00	0.00	20.40	36.10	0.06	3.596306876	22.24489796
Y	Y	u-6	ulsan	64.00	30	female	6	college and more	3000 KRW and more	1.00	1.00	2.00	3.00	6.00	11.70	15.90	0	4.0076515	29.76530612
Y	Y	u-7	ulsan	64.00	32	female	10	college and more	3000 KRW and more	2.00	1.00	1.00	20.00	2.00	9.50	11.70	2.13	3.844215466	33.84693878
Y	Y	u-8	ulsan	51.00	38	female	6	high school and less	3000 KRW and more	2.00	1.00	1.00	5.00	2.00	16.50	7.80	0.48	3.829320547	33.31122449
Y	Y	u-9	ulsan	64.00	35	female	4	college and more	under 3000 KRW	2.00	1.00	2.00	0.00	3.00	8.20	13.90	1.78	2.601226994	35.4193038
Y	Y	u-10	ulsan	65.00	40	female	3	college and more	3000 KRW and more	1.00	1.00	2.00	5.00	0.00	12.30	12.10	0.08	3.925729443	21.05102041
Y	Y	u-11	ulsan	57.00	44	female	3	college and more	3000 KRW and more	1.00	1.00	1.00	6.00	1.00	15.40	13.30	0.08	3.604774536	27.1122449
Y	Y	u-12	ulsan	43.00	52	female	7	high school and less	3000 KRW and more	2.00	1.00	1.00	12.00	7.00	18.60	21.80	0	3.887267905	17.89285714
Y	Y	u-13	ulsan	69.00	37	female	7	college and more	3000 KRW and more	2.00	1.00	2.00	5.00	0.00	11.20	17.40	1.67	4.056110998	45.75510204
Y	Y	u-14	ulsan	68.00	34	female	3	high school and less	3000 KRW and more	2.00	1.00	2.00	5.00	6.00	11.90	11.40	0.56	3.272189349	20.00510204
Y	Y	u-15	ulsan	67.00	36	female	5	college and more	3000 KRW and more	2.00	1.00	1.00	3.00	8.00	16.80	16.10	0.06	4.089879616	39.09693878
Y	Y	u-16	ulsan	43.00	58	female	7	high school and less	under 3000 KRW	2.00	1.00	2.00	19.00	0.00	14.50	12.70	2.1	3.465721281	5.704081633
Y	Y	u-17	ulsan	49.00	55	female	7	college and more	3000 KRW and more	1.00	1.00	2.00	18.00	0.00	18.90	12.40	0.5	3.276270149	10.95918367
Y	Y	u-18	ulsan	50.00	54	female	6	high school and less	3000 KRW and more	2.00	1.00	2.00	7.00	7.00	8.50	16.20	0.48	3.558763518	8.076530612
Y	Y	u-19	ulsan	45.00	52	female	7	high school and less	3000 KRW and more	2.00	1.00	2.00	1.00	4.00	13.70	19.10	0.24	3.713629871	11.96938776
Y	Y	u-20	ulsan	57.00	51	female	6	college and more	3000 KRW and more	1.00	1.00	1.00	13.00	0.00	15.00	14.20	0	3.428381963	12.50510204
Y	Y	u-21	ulsan	53.00	34	female	8	high school and less	3000 KRW and more	2.00	1.00	1.00	0.00	9.00	9.80	24.80	0.2	3.528667619	24.80612245
Y	Y	u-22	ulsan	49.00	33	female	7	college and more	3000 KRW and more	2.00	1.00	2.00	3.00	0.00	7.90	8.70	0.02	3.321465007	75.31632653
Y	Y	u-23	ulsan	49.00	42	female	6	999	3000 KRW and more	2.00	1.00	1.00	18.00	0.00	7.60	16.00	1.44	3.289328708	8.892857143
Y	Y	u-24	ulsan	51.00	43	female	6	college and more	3000 KRW and more	1.00	1.00	2.00	10.00	0.00	17.50	16.60	0.04	3.314935727	16.76530612
Y	Y	u-25	ulsan	77.00	39	female	8	college and more	3000 KRW and more	2.00	1.00	1.00	8.00	3.00	8.80	29.00	0.5	3.25351969	21.23469388
Y	Y	u-26	ulsan	70.00	29	female	3	college and more	3000 KRW and more	2.00	1.00	2.00	7.00	0.00	17.10	12.40	0.84	2.319347064	30.843
Y	Y	u-27	ulsan	70.00	45	female	2	high school and less	3000 KRW and more	2.00	1.00	2.00	2.00	0.00	12.30	6.90	0.02	3.966231381	20.20408163
Y	Y	u-28	ulsan	70.00	51	female	4	college and more	3000 KRW and more	2.00	1.00	2.00	5.00	0.00	7.80	15.10	0.4	3.486227301	14.55102041
Y	Y	u-29	ulsan	60.00	42	female	6	high school and less	3000 KRW and more	2.00	1.00	1.00	5.00	1.00	17.80	21.60	1.68	3.986941441	39.21938776
Y	Y	u-30	ulsan	63.00	21	female	7	college and more	3000 KRW and more	2.00	1.00	2.00	3.00	14.00	24.80	17.90	0.02	5.442460722	62.3622449
Y	Y	u-31	ulsan	63.00	79	female	5	high school and less	3000 KRW and more	2.00	1.00	2.00	3.00	26.00	7.50	35.80	0.28	3.654050194	21.19387755
Y	Y	u-32	ulsan	45.00	38	female	7	high school and less	3000 KRW and more	1.00	1.00	1.00	1.00	1.00	9.50	14.10	4.21	2.573948291	32.02373418
Y	Y	u-33	ulsan	52.00	56	female	2	high school and less	3000 KRW and more	1.00	1.00	1.00	20.00	1.00	10.20	14.20	0	6.116302795	42.15816327
Y	Y	u-34	ulsan	52.00	52	female	6	high school and less	3000 KRW and more	2.00	1.00	1.00	24.00	0.00	10.10	27.70	0.49	3.599673536	8.290816327
Y	Y	u-35	ulsan	53.00	47	female	2	college and more	3000 KRW and more	2.00	1.00	2.00	5.00	0.00	7.10	15.50	0.02	4.578759437	44.00510204
Y	Y	u-36	ulsan	56.00	40	female	6	college and more	3000 KRW and more	1.00	1.00	1.00	6.00	1.00	7.60	15.30	2.19	3.49908182	62.90816327
Y	Y	u-37	ulsan	66.00	45	female	8	high school and less	3000 KRW and more	1.00	1.00	2.00	10.00	0.00	9.30	9.70	0.1	4.131809835	18.66836735
Y	Y	u-38	ulsan	66.00	42	female	5	college and more	3000 KRW and more	2.00	1.00	1.00	5.00	1.00	8.20	19.30	0.16	3.787900428	26.60714286
Y	Y	u-39	ulsan	68.00	44	female	2	high school and less	3000 KRW and more	1.00	1.00	1.00	12.00	0.00	5.00	14.60	1.53	3.807692308	13.72959184
Y	Y	u-40	ulsan	67.00	46	female	3	high school and less	3000 KRW and more	2.00	1.00	1.00	4.00	0.00	23.00	20.00	0.08	4.116200775	17.44387755
Y	Y	u-41	ulsan	87.00	53	female	2	high school and less	3000 KRW and more	1.00	1.00	1.00	18.00	0.00	11.30	22.10	1.86	3.265354009	9.183673469
Y	Y	u-42	ulsan	54.00	53	female	2	high school and less	3000 KRW and more	2.00	1.00	1.00	17.00	0.00	13.60	6.80	0.61	3.164660273	4.454081633
Y	Y	u-43	ulsan	58.00	36	female	7	college and more	3000 KRW and more	2.00	1.00	2.00	8.00	1.00	7.80	25.00	0.02	4.206590492	21.41836735
Y	Y	u-44	ulsan	56.00	49	female	2	high school and less	3000 KRW and more	2.00	1.00	2.00	15.00	0.00	10.20	17.30	0.16	4.286472149	22.15306122
Y	Y	u-45	ulsan	60.00	1	male	1	high school and less	under 3000 KRW	1.00	1.00	1.00	17.00	1.00	7.30	6.50	0.82	3.454601102	9.423469388
Y	Y	u-46	ulsan	53.00	34	female	0	college and more	under 3000 KRW	1.00	1.00	1.00	6.00	7.00	9.20	13.90	0.3	3.457253622	9.959183673
Y	Y	u-47	ulsan	60.00	45	female	6	college and more	3000 KRW and more	1.00	1.00	2.00	17.00	2.00	17.90	23.80	23.82	3.897775964	13.41326531
Y	Y	u-48	ulsan	67.00	57	female	7	high school and less	3000 KRW and more	1.00	1.00	1.00	5.00	1.00	14.70	24.20	0.02	3.650275454	15.6377551
Y	Y	u-49	ulsan	66.00	30	female	2	college and more	3000 KRW and more	2.00	1.00	2.00	3.00	0.00	17.40	12.60	0.61	3.671597633	91.14795918
Y	Y	u-50	ulsan	66.00	53	female	5	high school and less	3000 KRW and more	2.00	1.00	2.00	3.00	1.00	13.30	19.00	0.06	4.25321363	117.7908163
Y	Y	u-51	ulsan	64.00	38	female	7	college and more	3000 KRW and more	2.00	1.00	2.00	1.00	1.00	9.60	12.20	0.64	3.24209345	22.69387755
Y	Y	u-52	ulsan	55.00	52	female	6	high school and less	3000 KRW and more	2.00	1.00	1.00	3.00	2.00	18.60	16.00	0.68	3.343501326	12.12755102
Y	Y	u-53	ulsan	53.00	51	female	4	high school and less	3000 KRW and more	2.00	1.00	1.00	2.00	0.00	12.10	31.70	0.42	3.333809427	18.89795918
Y	Y	u-54	ulsan	59.00	41	female	1	college and more	3000 KRW and more	2.00	1.00	1.00	6.00	0.00	15.10	13.50	0.12	4.095184656	16.13265306
Y	Y	u-55	ulsan	51.00	48	female	8	high school and less	3000 KRW and more	1.00	1.00	1.00	7.00	1.00	12.10	9.70	0.96	4.26015099	40.15306122
Y	Y	u-56	ulsan	56.00	52	female	2	high school and less	3000 KRW and more	2.00	1.00	1.00	20.00	0.00	17.90	15.40	0.42	3.517649459	22.92346939
Y	Y	u-57	ulsan	62.00	54	female	8	high school and less	3000 KRW and more	2.00	1.00	1.00	10.00	1.00	25.80	25.00	0.16	4.566108957	27.70408163

Y	Y	u-58	ulsan	74.00	45	female	7	college and more	3000 KRW and more	1.00	1.00	1.00	11.00	1.00	15.20	8.70	0.3	3.49255254	15.11734694
Y	Y	u-59	ulsan	55.00	40	female	8	college and more	3000 KRW and more	1.00	1.00	1.00	3.00	1.00	17.70	14.30	0.18	4.25688635	13.52040816
Y	Y	u-60	ulsan	55.00	52	female	5	college and more	3000 KRW and more	2.00	1.00	1.00	14.00	1.00	16.60	15.30	0.48	3.552642318	16.2244898
Y	Y	u-61	ulsan	55.00	20	female	6	college and more	3000 KRW and more	2.00	1.00	2.00	14.00	1.00	20.70	8.40	0.6	3.711691492	23.92857143
Y	Y	u-62	ulsan	72.00	49	female	2	high school and less	3000 KRW and more	1.00	1.00	1.00	7.00	0.00	8.60	6.10	0.12	4.443276882	26.60204082
Y	Y	u-63	ulsan	73.00	73	female	7	college and more	3000 KRW and more	2.00	1.00	1.00	25.00	0.00	17.10	28.50	0.6	3.427769843	12.62755102
Y	Y	u-64	ulsan	63.00	48	female	8	college and more	under 3000 KRW	1.00	1.00	2.00	21.00	0.00	15.10	9.20	0	4.126810855	38.17857143
Y	Y	u-65	ulsan	67.00	36	female	2	high school and less	3000 KRW and more	2.00	1.00	2.00	2.00	0.00	14.30	14.90	0.56	3.381452765	6.37244898
Y	Y	u-66	ulsan	71.00	50	female	6	high school and less	3000 KRW and more	1.00	1.00	1.00	17.00	3.00	9.50	17.70	0.16	3.790042848	13.23979592
Y	Y	u-67	ulsan	69.00	44	female	7	high school and less	3000 KRW and more	1.00	1.00	2.00	18.00	1.00	20.20	19.20	0.04	3.560497858	27.33163265
Y	Y	u-68	ulsan	70.00	52	female	9	high school and less	3000 KRW and more	1.00	1.00	1.00	17.00	1.00	8.60	24.80	0	4.311671088	26.40816327
Y	Y	u-69	ulsan	70.00	23	female	5	college and more	3000 KRW and more	2.00	1.00	2.00	17.00	0.00	16.60	10.70	0.01	4.385227505	29.0255102
Y	Y	u-70	ulsan	68.00	50	female	5	high school and less	3000 KRW and more	2.00	1.00	1.00	17.00	1.00	12.10	20.40	1.63	3.423280963	16.54081633
Y	Y	u-71	ulsan	66.00	46	female	2	high school and less	3000 KRW and more	2.00	1.00	1.00	14.00	0.00	13.00	22.20	0	3.782493369	30.07142857
Y	Y	u-72	ulsan	67.00	45	female	1	college and more	3000 KRW and more	2.00	1.00	1.00	17.00	4.00	17.50	29.40	0.18	3.180983473	19.6377551
Y	Y	u-73	ulsan	68.00	55	female	7	high school and less	3000 KRW and more	2.00	1.00	2.00	17.00	1.00	8.70	24.20	0.04	3.295449908	15.41836735
Y	Y	u-74	ulsan	67.00	47	female	4	high school and less	3000 KRW and more	2.00	1.00	2.00	19.00	1.00	7.60	16.80	0.24	4.056723118	11.69897959
Y	Y	u-75	ulsan	52.00	43	female	0	college and more	under 3000 KRW	1.00	1.00	1.00	5.00	0.00	9.50	27.90	0.35	4.856253826	43.75510204
Y	Y	u-76	ulsan	66.00	37	female	8	college and more	3000 KRW and more	1.00	1.00	2.00	8.00	1.00	10.50	7.40	0.56	3.357580086	14.49489796
Y	Y	u-77	ulsan	65.00	48	female	5	high school and less	3000 KRW and more	2.00	1.00	1.00	2.00	1.00	15.90	20.40	0.36	3.23811467	7.790816327
Y	Y	u-78	ulsan	70.00	39	female	8	college and more	3000 KRW and more	2.00	1.00	1.00	6.00	0.00	11.40	17.80	0.04	3.413385023	23.16836735
Y	Y	u-79	ulsan	71.00	27	female	4	college and more	3000 KRW and more	1.00	1.00	2.00	10.00	12.00	23.60	12.40	0.02	4.285145889	124.3826531
Y	Y	u-80	ulsan	71.00	54	female	6	college and more	3000 KRW and more	2.00	1.00	1.00	10.00	19.00	16.20	21.90	1.92	4.195368292	29.31122449
Y	Y	u-81	ulsan	71.00	60	female	5	high school and less	3000 KRW and more	2.00	1.00	2.00	9.00	9.00	6.20	18.40	0.04	3.443684962	23.2244898
Y	Y	u-82	ulsan	71.00	33	female	6	college and more	3000 KRW and more	2.00	1.00	2.00	9.00	12.00	26.90	6.40	0.04	3.693021832	33.81632653
Y	Y	u-83	ulsan	66.00	45	female	5	college and more	3000 KRW and more	2.00	1.00	1.00	3.00	0.00	17.30	21.70	1.04	2.637489045	32.9414557
Y	Y	u-84	ulsan	52.00	40	female	4	high school and less	3000 KRW and more	1.00	1.00	1.00	4.00	1.00	6.00	10.80	0.04	3.717302591	12.28571429
Y	Y	u-85	ulsan	59.00	35	female	3	college and more	3000 KRW and more	2.00	1.00	1.00	27.00	0.00	9.20	13.30	0.02	3.402468884	44.81632653
Y	Y	u-86	ulsan	52.00	30	female	6	high school and less	under 3000 KRW	2.00	1.00	2.00	0.00	22.00	25.50	16.50	0.92	3.678432973	20.39795918
Y	Y	u-87	ulsan	76.00	53	female	7	college and more	3000 KRW and more	2.00	1.00	2.00	5.00	0.00	13.50	10.80	0.06	6.482860641	228.9540816
Y	Y	u-88	ulsan	50.00	31	female	4	high school and less	under 3000 KRW	2.00	2.00	2.00	1.00	14.00	6.50	3.90	0	3.192817792	39.04081633
Y	Y	u-89	ulsan	56.00	28	female	8	college and more	under 3000 KRW	1.00	1.00	2.00	1.00	9.00	16.10	23.20	0.14	4.456947562	46.08673469
Y	Y	u-90	ulsan	46.00	56	female	8	high school and less	3000 KRW and more	2.00	1.00	1.00	19.00	0.00	15.20	4.80	3.09	4.415753725	61.64082278
Y	Y	u-91	ulsan	47.00	40	female	8	college and more	3000 KRW and more	2.00	1.00	2.00	3.00	4.00	14.90	20.00	0	3.412466844	38.67857143
Y	Y	u-92	ulsan	69.00	34	female	6	college and more	3000 KRW and more	1.00	1.00	2.00	1.00	3.00	7.60	16.40	0.1	4.00877372	24.05102041
Y	Y	u-93	ulsan	51.00	50	female	3	college and more	3000 KRW and more	1.00	1.00	1.00	15.00	0.00	7.70	14.50	0.2	3.769026729	8.265306122
Y	Y	u-94	ulsan	51.00	56	female	5	high school and less	3000 KRW and more	2.00	1.00	2.00	17.00	3.00	15.50	19.10	0.04	4.265354009	35.66836735
Y	Y	u-95	ulsan	51.00	53	female	3	high school and less	3000 KRW and more	2.00	1.00	1.00	15.00	0.00	11.50	14.00	0.16	3.404713324	15.69387755
Y	Y	u-96	ulsan	52.00	45	female	2	college and more	3000 KRW and more	2.00	1.00	1.00	10.00	0.00	10.20	17.90	0.62	3.481024281	20.98979592
Y	Y	u-97	ulsan	69.00	54	female	6	high school and less	3000 KRW and more	1.00	1.00	1.00	16.00	2.00	22.30	28.00	0.02	3.912262804	21.23979592
Y	Y	u-98	ulsan	69.00	25	female	7	college and more	3000 KRW and more	2.00	1.00	2.00	16.00	0.00	27.80	12.70	0.06	4.24250153	41.96938776
Y	Y	u-99	ulsan	70.00	49	female	10	college and more	3000 KRW and more	2.00	1.00	1.00	10.00	1.00	12.90	44.50	1.4	4.129157315	19.69897959
Y	Y	u-100	ulsan	45.00	46	female	6	high school and less	3000 KRW and more	1.00	1.00	1.00	23.00	4.00	13.30	21.10	0.2	4.427973883	15.69387755
Y	Y	u-101	ulsan	49.00	74	female	8	high school and less	3000 KRW and more	2.00	1.00	2.00	27.00	3.00	22.70	41.90	0.02	3.409814324	10.41326531
Y	Y	u-102	ulsan	49.00	51	female	3	high school and less	under 3000 KRW	1.00	1.00	1.00	8.00	0.00	10.80	15.80	0.6	3.322791267	21.07653061
Y	Y	u-103	ulsan	44.00	48	female	8	high school and less	under 3000 KRW	1.00	2.00	2.00	4.00	4.00	16.50	4.40	0	3.75107121	23.75510204
Y	Y	u-104	ulsan	72.00	51	female	7	high school and less	3000 KRW and more	1.00	1.00	2.00	5.00	6.00	19.10	13.10	0.7	3.737400531	23.57653061
Y	Y	u-105	ulsan	64.00	60	female	5	high school and less	under 3000 KRW	2.00	1.00	2.00	3.00	0.00	19.40	24.60	4.05	2.198290973	27.12341772
Y	Y	u-106	ulsan	55.00	71	female	8	high school and less	under 3000 KRW	2.00	1.00	1.00	5.00	0.00	12.10	28.90	1.16	2.28878177	27.78164557
Y	Y	u-107	ulsan	70.00	53	female	7	college and more	3000 KRW and more	1.00	1.00	1.00	4.00	0.00	6.90	11.20	3.27	2.227760736	29.35601266
Y	Y	u-108	ulsan	59.00	39	female	8	college and more	3000 KRW and more	2.00	1.00	1.00	1.00	6.00	12.30	32.50	0.02	3.609365436	24.03571429
Y	Y	u-109	ulsan	56.00	31	female	8	college and more	3000 KRW and more	1.00	1.00	2.00	1.00	10.00	8.60	8.80	0.2	3.660885534	35.66836735
Y	Y	u-110	ulsan	61.00	34	female	6	college and more	3000 KRW and more	1.00	1.00	1.00	5.00	6.00	9.40	14.80	0.88	2.546121823	31.29746835
Y	Y	u-111	ulsan	57.00	50	female	5	high school and less	3000 KRW and more	2.00	1.00	2.00	20.00	24.00	21.90	8.90	0	3.222199551	21.5255102
Y	Y	u-112	ulsan	57.00	23	female	4	high school and less	3000 KRW and more	2.00	1.00	2.00	20.00	61.00	21.40	14.30	0.82	3.609569476	27.48469388
Y	Y	u-113	ulsan	53.00	61	female	8	high school and less	3000 KRW and more	2.00	1.00	1.00	33.00	0.00	28.70	15.80	0.06	3.482860641	16.6377551
Y	Y	u-114	ulsan	46.00	28	female	7	college and more	3000 KRW and more	2.00	1.00	1.00	2.00	68.00	29.00	10.60	0.22	3.475923281	13.57653061
Y	Y	u-115	ulsan	62.00	60	female	7	college and more	3000 KRW and more	2.00	1.00	1.00	18.00	8.00	18.30	36.00	0.02	3.414099163	25

Y	Y	u-116	ulsan	61.00	60	female	7	high school and less	3000 KRW and more	1.00	1.00	1.00	1.00	15.00	12.20	27.90	0.02	3.371556825	21.89795918
Y	Y	u-117	ulsan	73.00	57	female	5	high school and less	3000 KRW and more	2.00	1.00	1.00	3.00	0.00	20.30	26.60	0.04	3.290552948	17.05102041
Y	Y	u-118	ulsan	63.00	58	female	7	high school and less	under 3000 KRW	1.00	1.00	1.00	3.00	7.00	11.30	14.90	0.5	3.339012446	25.08163265
Y	Y	u-119	ulsan	66.00	44	female	2	college and more	under 3000 KRW	1.00	1.00	1.00	3.00	4.00	17.00	17.60	0.7	3.384003265	19.67346939
Y	Y	u-120	ulsan	49.00	55	female	7	high school and less	3000 KRW and more	2.00	1.00	1.00	22.00	0.00	12.70	16.70	0.04	3.24954091	10.94387755
Y	Y	u-121	ulsan	49.00	26	female	3	college and more	under 3000 KRW	1.00	1.00	1.00	22.00	0.00	11.70	11.10	0.08	3.336972047	9.693877551
Y	Y	u-122	ulsan	66.00	45	female	5	college and more	3000 KRW and more	2.00	1.00	2.00	16.00	5.00	15.80	14.00	0	3.848704346	19.06632653
Y	Y	u-123	ulsan	65.00	35	female	6	college and more	3000 KRW and more	1.00	1.00	2.00	1.00	5.00	16.00	15.30	0.26	4.030605999	32.80102041
Y	Y	u-124	ulsan	66.00	42	female	9	college and more	3000 KRW and more	1.00	1.00	2.00	0.00	7.00	17.50	28.10	0.62	3.946337482	20.77040816
Y	Y	u-125	ulsan	46.00	48	female	7	college and more	3000 KRW and more	2.00	1.00	2.00	1.00	0.00	9.60	15.10	0.06	3.437563762	7.484693878
Y	Y	u-126	ulsan	46.00	33	female	4	college and more	3000 KRW and more	1.00	1.00	2.00	1.00	4.00	7.50	9.30	0.5	3.50846766	112.8010204
Y	Y	u-127	ulsan	53.00	43	female	4	high school and less	3000 KRW and more	1.00	1.00	1.00	3.00	1.00	13.10	7.20	0.02	6.125484595	92.59693878
Y	Y	u-128	ulsan	61.00	49	female	5	high school and less	3000 KRW and more	1.00	1.00	1.00	12.00	1.00	11.60	11.00	0.12	3.73882881	29.92346939
Y	Y	s-1	seoul	65.80	30	female	5	college and more	under 3000 KRW	2.00	1.00	2.00	2.00	8.00	19.50	15.30	12.39	1.972173532	17.66297468
Y	Y	s-2	seoul	52.10	51	female	5	college and more	under 3000 KRW	2.00	1.00	2.00	4.00	6.00	12.50	20.30	0.4	3.39328708	25.3214285
Y	Y	s-3	seoul	62.80	25	female	6	college and more	3000 KRW and more	2.00	1.00	2.00	12.00	7.00	14.00	13.50	2.29	1.755039439	19.1693038
Y	Y	s-4	seoul	62.80	54	female	8	college and more	3000 KRW and more	2.00	1.00	2.00	12.00	5.00	19.40	30.30	0.86	2.23115688	40.09335443
Y	Y	s-5	seoul	56.10	46	female	8	high school and less	under 3000 KRW	999.00	1.00	2.00	5.00	9.00	21.80	5.20	3.29	3.9765354	45.3979591
Y	Y	s-6	seoul	44.90	41	female	5	999	999	999.00	1.00	2.00	1.00	3.00	7.80	13.20	0.44	3.24984697	25.132653
Y	Y	s-7	seoul	47.10	44	female	5	high school and less	3000 KRW and more	999.00	1.00	2.00	999.00	4.00	11.40	18.80	3.22	1.752373649	15.97679325
Y	Y	s-8	seoul	37.50	44	female	8	high school and less	under 3000 KRW	999.00	1.00	2.00	30.00	0.00	13.50	25.00	4.19	1.955302366	17.48417722
Y	Y	s-9	seoul	46.00	34	female	8	college and more	999	999.00	1.00	2.00	0.00	0.00	11.40	26.80	1.44	3.30901856	19.9387755
Y	Y	s-10	seoul	35.50	59	female	8	college and more	under 3000 KRW	999.00	1.00	2.00	13.00	31.00	8.20	18.90	0.5	3.39114466	26.8622449
Y	Y	s-11	seoul	44.90	55	female	2	high school and less	under 3000 KRW	999.00	1.00	1.00	28.00	4.00	12.90	9.60	3.52	1.699094362	18.34493671
Y	Y	s-12	seoul	59.30	41	female	5	college and more	under 3000 KRW	999.00	1.00	1.00	10.00	10.00	14.60	27.60	0.87	3.33003468	12.7806122
Y	Y	s-13	seoul	41.80	63	female	8	high school and less	999	999.00	1.00	1.00	4.00	7.00	19.50	16.90	0.54	3.56110997	14.9540816
Y	Y	s-14	seoul	61.80	59	female	5	high school and less	999	999.00	1.00	1.00	3.00	11.00	13.90	8.00	0.66	3.35829422	24.1377551
Y	Y	s-15	seoul	53.20	57	female	9	college and more	999	999.00	1.00	2.00	5.00	6.00	20.20	6.60	0.54	3.44225668	23.4183673
Y	Y	s-16	seoul	58.90	42	female	4	college and more	3000 KRW and more	999.00	1.00	1.00	3.00	7.00	10.50	11.90	3.95	1.593229623	19.69831224
Y	Y	s-17	seoul	57.40	40	female	7	college and more	3000 KRW and more	999.00	1.00	2.00	3.00	0.00	11.40	7.20	0.56	3.40073454	30.382653
Y	Y	s-18	seoul	50.40	36	female	10	college and more	3000 KRW and more	999.00	1.00	2.00	3.00	5.00	19.70	21.40	0.68	1.866564417	20.67879747
Y	Y	s-19	seoul	47.90	20	female	5	college and more	under 3000 KRW	999.00	1.00	2.00	1.00	1.00	15.60	8.00	0.5	3.74372577	33.2295918
Y	Y	s-20	seoul	47.90	23	female	5	high school and less	under 3000 KRW	999.00	1.00	2.00	1.00	57.00	11.90	11.50	3.03	1.609881683	14.76740506
Y	Y	s-21	seoul	56.80	49	female	4	college and more	3000 KRW and more	999.00	1.00	1.00	20.00	1.00	8.60	16.60	5.86	1.589249197	21.40981013
Y	Y	s-22	seoul	56.80	21	female	7	high school and less	3000 KRW and more	999.00	1.00	2.00	999.00	0.00	8.00	24.80	0.98	1.899430324	15.85126582
Y	Y	s-23	seoul	49.70	60	female	8	high school and less	999	999.00	1.00	2.00	10.00	19.00	12.70	21.60	0.32	3.10518261	15.6785714
Y	Y	s-24	seoul	65.90	45	female	6	high school and less	3000 KRW and more	999.00	1.00	1.00	9.00	56.00	15.20	21.90	0.46	1.864263804	20.41297468
Y	Y	s-25	seoul	38.90	25	female	7	college and more	under 3000 KRW	999.00	1.00	2.00	26.00	11.00	15.30	13.80	8.74	1.950810692	21.06170886
Y	Y	s-26	seoul	69.90	44	female	10	high school and less	under 3000 KRW	999.00	1.00	2.00	15.00	0.00	12.40	7.80	5.76	1.707493427	14.98259494
Y	Y	s-27	seoul	45.20	57	female	3	high school and less	under 3000 KRW	999.00	1.00	2.00	8.00	3.00	6.70	12.00	1.42	1.628177038	22.9556962
Y	Y	s-28	seoul	66.30	62	female	3	college and more	3000 KRW and more	999.00	1.00	2.00	12.00	2.00	5.70	22.80	0.24	3.88237094	35.382653
Y	Y	s-29	seoul	68.60	52	female	9	high school and less	3000 KRW and more	999.00	1.00	1.00	7.00	6.00	20.20	12.60	0.36	3.25453989	14.127551
Y	Y	s-30	seoul	61.00	56	female	5	college and more	under 3000 KRW	999.00	1.00	1.00	10.00	15.00	15.70	4.30	2.76	2.126971954	44.63449367
Y	Y	s-31	seoul	72.50	41	female	10	high school and less	3000 KRW and more	999.00	1.00	2.00	0.00	12.00	8.00	20.20	4.61	1.793163891	18.75738397
Y	Y	s-32	seoul	71.30	32	female	5	college and more	3000 KRW and more	999.00	1.00	2.00	3.00	4.00	9.50	15.10	2.36	1.608567046	15.94303797
Y	Y	s-33	seoul	39.80	67	female	8	high school and less	under 3000 KRW	999.00	1.00	2.00	5.00	0.00	17.10	25.00	0.62	1.687445223	12.36867089
Y	Y	s-34	seoul	44.40	71	female	7	high school and less	under 3000 KRW	999.00	1.00	2.00	1.00	16.00	13.70	10.50	12.62	2.004601227	20.86550633
Y	Y	s-35	seoul	40.60	42	female	8	high school and less	under 3000 KRW	999.00	1.00	2.00	10.00	0.00	6.20	18.10	4.55	1.619412796	16.61708861
Y	Y	s-36	seoul	50.40	56	female	10	high school and less	under 3000 KRW	999.00	1.00	2.00	0.00	0.00	4.80	9.40	2	1.790644172	22.52531646
Y	Y	s-37	seoul	52.10	35	female	6	college and more	3000 KRW and more	999.00	1.00	2.00	3.00	0.00	13.90	15.50	0.6	1.885078878	17.02531646
Y	Y	s-38	seoul	42.00	51	female	9	high school and less	3000 KRW and more	999.00	1.00	2.00	6.00	0.00	13.10	10.90	1.64	1.72754163	20.53955696
Y	Y	s-39	seoul	42.00	53	female	8	high school and less	3000 KRW and more	999.00	1.00	2.00	21.00	2.00	6.70	5.40	5.44	1.829206836	23.48259494
Y	Y	s-40	seoul	49.40	38	female	5	college and more	3000 KRW and more	999.00	1.00	2.00	1.00	30.00	11.80	12.40	7.17	1.727906807	20.14820675
Y	Y	s-41	seoul	58.60	33	female	3	high school and less	under 3000 KRW	999.00	1.00	2.00	8.00	43.00	10.70	16.60	15.57	1.498794917	22.97943038
Y	Y	s-42	seoul	50.50	59	female	8	high school and less	under 3000 KRW	999.00	1.00	1.00	15.00	2.00	16.40	21.40	2.21	1.546450482	17.25474684
Y	Y	s-43	seoul	59.90	48	female	5	college and more	3000 KRW and more	999.00	1.00	1.00	2.00	1.00	13.80	15.70	0.82	3.3428892	14.3622449
Y	Y	s-44	seoul	70.60	47	female	7	high school and less	3000 KRW and more	999.00	1.00	1.00	0.00	999.00	13.70	12.70	0.1	3.3255458	8.22448979
